# Orai1 forms a signal complex with BK_C__a_ channel in mesenteric artery smooth muscle cells

**DOI:** 10.14814/phy2.12682

**Published:** 2016-01-12

**Authors:** Meihua Chen, Jie Li, Feifei Jiang, Jie Fu, Xianming Xia, Juan Du, Min Hu, Junhao Huang, Bing Shen

**Affiliations:** ^1^ Department of PhysiologyAnhui Medical UniversityHefeiAnhuiChina; ^2^ Department of PediatricsThe people's hospital of BozhouBozhouAnhuiChina; ^3^ Department of Gastroenterology and HepatologyThe Fourth Affiliated Hospital of Anhui Medical UniversityHefeiAnhuiChina; ^4^ Department of Sports and HealthGuangdong Provincial Key Laboratory of Sports and Health PromotionGuangzhou Sport UniversityGuangzhouGuangdongChina; ^5^ Central Laboratory of Molecular and Cellular Biology of School of Basic MedicineAnhui Medical UniversityHefeiAnhuiChina

**Keywords:** BK_C__a_, mesenteric artery, Orai1, store‐operated Ca^2+^ entry, vascular smooth muscle cells

## Abstract

Orai1, a specific nonvoltage‐gated Ca^2+^ channel, has been found to be one of key molecules involved in store‐operated Ca^2+^ entry (SOCE). Orai1 may associate with other proteins to form a signaling complex, which is essential for regulating a variety of physiological functions. In this study, we studied the possible interaction between Orai1 and large conductance Ca^2+^‐activated potassium channel (BK_C__a_). Using RNA interference technique, we demonstrated that the SOCE and its associated membrane hyperpolarization were markedly suppressed after knockdown of Orai1 with a specific Orai1 siRNA in rat mesenteric artery smooth muscle. Moreover, isometric tension measurements showed that agonist‐induced vasocontraction was increased after Orai1 was knocked down or the tissue was incubated with BK_C__a_ blocker iberiotoxin. Coimmunoprecipitation data revealed that BK_C__a_ and Orai1 could reciprocally pull down each other. In situ proximity ligation assay further demonstrated that Orai1 and BK_C__a_ are in close proximity. Taken together, these results indicate that Orai1 physically associates with BK_C__a_ to form a signaling complex in the rat mesenteric artery smooth muscle. Ca^2+^ influx via Orai1 stimulates BK_C__a_, leading to membrane hyperpolarization. This hyperpolarizing effect of Orai1‐BK_C__a_ coupling could contribute to reduce agonist‐induced membrane depolarization, therefore preventing excessive contraction of the rat mesenteric artery smooth muscle in response to contractile agonists.

## Introduction

Ca^2+^ ion is an important intracellular second messenger, regulating a plethora of cell functions such as secretion, transcription, growth, and apoptosis (Bootman and Berridge 1995; Berridge 1998, 2013; Genazzani and Thorn 2002; Lipskaia and Lompre 2004). One of the most common and ubiquitous pathways involved in modulating Ca^2+^ influx into cells is store‐operated Ca^2+^ entry (SOCE), which is mediated via specific plasma membrane ion channels in response to the depletion of Ca^2+^ content of intracellular Ca^2+^ stores (Gwack et al. 2007; Dominguez‐Rodriguez et al. 2012). The previous studies showed that knockdown of Orai1, one of the key components of SOCE, reduced SOCE activity (Li et al. 2011; Yang et al. 2012).

The Orai1 is a plasma membrane protein predicted to contain four TM segments (TM1 to TM4) with both N‐ and C‐termini located in the cytosol. Orai1 is widely expressed in many cell types, including vascular smooth muscle cells (VSMCs) and endothelial cells (Beech 2012; Berna‐Erro et al. 2012; Trebak 2012). Functionally, Orai1 activity is associated with vascular remodeling that relative to neointimal hyperplasia and angiogenesis. For example, Orai1 plays significant positive roles in migrating and proliferating behaviors of VSMCs. Inhibition of migration and proliferation has been observed after Orai1 knockdown by siRNA (Potier et al. 2009; Zhang et al. 2011).

Accumulated evidence suggests that Orai1 could interact with Ca^2+^‐activated potassium (K_Ca_) channels in modulating SOCE (Clarysse et al. 2014; Lin et al. 2014). Recently, one study showed that Orai1 forms complex with SK3 in lipid rafts to control constitutive Ca^2+^ entry and cancer cell migration, as well as bone metastasis (Chantome et al. 2013). Furthermore, our study also demonstrated that Orai1 could form a signaling complex with SK3, modulating SOCE and its associated membrane hyperpolarization in gallbladder smooth muscle (Song et al. 2015). However, whether Orai1 interacts with BK_Ca_ in VSMCs is still unknown. In this study, we investigated possible interaction between Orai1 and BK_Ca_ in VSMCs of rat mesenteric arteries. Our results showed that Orai1 physically associates with BK_Ca_ to form a signaling complex and that Ca^2+^ influx through Orai1 activates BK_Ca_ to induce membrane hyperpolarization. This hyperpolarizing effect of Orai1‐BK_Ca_ coupling may contribute to prevent excessive contraction of smooth muscle in response to contractile agonists.

## Materials and Methods

### Materials

Phenylephrine (Phe) and iberiotoxin (IbTX) were purchased from Sigma‐Aldrich. Thapsigargin (TG) and endothelin 1 (ET‐1) were obtained from Calbiochem. Anti‐Orai1 (sc‐74778) primary antibody was purchased from Santa Cruz. Anti‐BK_Ca_ (APC‐107) primary antibody was from Alomone Lab. Orai1 specific siRNA for rat was obtained from Invitrogen. The sequence is as follows: CAACAGCAAUCCGGAGCUU (Potier et al. 2009).

### Cell culture

All animal experiments were conducted in accordance with NIH publication no. 8523 and were approved by the Animal Experimentation Ethics Committee of Anhui Medical University. Primary cultured VSMCs were isolated from Sprague–Dawley rats. Briefly, mesenteric artery was dissected. After rubbing off endothelial layer, smooth muscle layer was peeled off and then digested with 0.2% collagenase type IA and 0.9% papain for 1 h. The dispersed VSMCs were cultured in Dulbecco modified Eagle's medium (DMEM) containing 10% fetal bovine serum (FBS), 100 U/mL penicillin and 100 *μ*g/mL streptomycin for 5 to 7 days before experimental use. Cells were grown at 37°C in a 5% CO_2_ humidified incubator. The first passage was used in all experiments.

### Membrane potential measurement

Membrane potential was measured as previously described (Kwan et al. 2009). Briefly, primary cultured VSMCs were loaded with 100 nmol/L of potentiometric fluorescence dye bis‐oxonol [DiBAC_4_(3)] at 37°C for 10 min. Cells were incubated with/without IbTX (50 nmol/L) for 10 min, or with scrambled siRNA or Orai1 siRNA for 24 h. Cells were treated with 4 *μ*mol/L TG in the dark for 8 to 15 min in a Ca^2+^‐free physiological saline solution (0Ca^2+^‐PSS), which contained (in mmol/L): 140 NaCl, 5 KCl, 1 MgCl_2_, 10 glucose, 0.2 EGTA, 5 Hepes, pH 7.4. SOCE was then initiated by applying 1 mmol/L extracellular Ca^2+^, resulting in a marked membrane hyperpolarization. Changes in fluorescence were measured by Nikon Diaphot inverted microscope.

### Immunoprecipitation and immunoblots

Immunoprecipitation and immunoblots were performed as previously described (Kwan et al. 2004). In brief, smooth muscle layer was peeled off from the adventitial layer with forceps, followed by homogenization. The proteins were extracted from smooth muscle cell lysates with detergent extracted buffer, which contained 1% (vol/vol) Nonidet P‐40, 150 mmol/L NaCl, 20 mmol/L Tris‐HCl, pH 8.0, with the addition of protease inhibitor cocktail tablets. 800 *μ*g of extracted proteins was then incubated with 3 *μ*g of anti‐Orai1 or anti‐BK_Ca_ antibody on a rocking platform overnight at 4°C. Protein A agarose was then applied, followed by a further incubation at 4°C for 3 h. The immunoprecipitates were washed with saline for three times and then resolved on 8% SDS‐PAGE gel. The proteins were then transferred to a PVDF membrane using a semidry transfer system (Bio‐Rad). The membrane carrying the transferred proteins was incubated at 4°C overnight with the primary antibody at 1:250 dilution in PBST buffer containing 0.1% Tween 20 and 5% nonfat dry milk. Immunodetection was accomplished using horseradish peroxidase‐conjugated secondary antibody. Antibody binding was detected by the ECL system.

### Mesenteric artery tension measurement

Mesenteric artery tension measurement was performed as previously reported (Kwan et al. 2009). Briefly, segments of the tertiary branches of rat mesenteric artery (2 mm long) were dissected in a Petri dish filled with ice‐cold Krebs solution (composition in mM: 118 NaCl, 4.7 KCl, 2.5 CaCl_2_, 1.2 KH_2_PO_4_, 1.2 MgSO_4_ (7 H_2_O), 25.2 NaHCO_3_, and 11.1 glucose) oxygenated with a gas mixture of 95% O_2_ and 5% CO_2_. The segments were mounted in a DMT myograph (model 610M; Danish Myo Technology, Aarhus, Denmark) under a normalized tension as previously described (Cheng et al. 2008). After the equilibration period, the contractile function of the vessel was tested by replacing the Krebs solution with 60 mmol/L K^+^ solution (60 mmol/L high K^+^ solution was prepared by substituting NaCl with an equimolar amount of KCl). After the washout, the rings were challenged with 1 *μ*mol/L Phe to test their contractile responses and subsequently exposed to 1 *μ*mol/L acetylcholine to verify endothelial integrity. The contractile response to Phe (10^−7.5^–10^−5^ mol/L) or ET‐1 (10^‐10^‐10^‐8^) were obtained by cumulatively adding agonists into the bath with or without the pretreatment of IbTX (50 *μ*mol/L) for 10 min. For the Orai1 knockdown experiment, mesenteric artery was incubated with Orai1 siRNA or scrambled siRNA for 24 h before experiments.

### [Ca^2+^]_i_ measurement

Cytosolic Ca^2+^ ([Ca^2+^]_i_) was measured as previously described (Shen et al. 2011). In brief, cells were incubated with 10 *μ*mol/L Fluo‐8/AM and 0.02% pluronic F‐127 (Invitrogen, Carlsbad, CA) for 40 min in the dark at 37°C. Ca^2+^ stores were depleted by treating cells with 4 *μ*mol/L TG for 10 min in 0Ca^2+^‐PSS. Ca^2+^ influx was initiated by applying 1 mmol/L extracellular Ca^2+^. Cells were pretreated with scrambled siRNA or Orai1 siRNA for 24 h before experiments. Fluorescence signal was recorded by Leica TCS SP5 confocal laser system. Changes in [Ca^2+^]_i_ were displayed as the ratio of fluorescence relative to the intensity before applying extracellular Ca^2+^ (F1/F0).

### In situ proximity ligation assay (PLA)

Interaction of Orai1 with BK_Ca_ was detected by using in situ PLA kit Duolink (Sigma‐Aldrich, St. Louis, MO), following the manufacturer's instructions. Briefly, VSMCs were freshly isolated from mesenteric arteries and attached on coverslips. The cells were fixed and permeabilized. After blocked with Duolink blocking solution, VSMCs were incubated with anti‐Orai1 and anti‐BK_Ca_ (1:40, each) antibodies overnight at 4°C in Duolink antibody diluent. Negative control slides were incubated with anti‐Orai1 antibody alone. Then cells were washed with physiological saline solution and incubated with Duolink secondary antibodies conjugated with oligonucleotides (anti‐goat PLA probe Plus, Cat. DUO92003 and anti‐rabbit PLA probe Minus, Cat. DUO92005) in a preheated humidity chamber for 1 h at 37°C. Subsequently, cells were incubated with a ligation solution containing two oligonucleotides and one ligase. The oligonucleotides hybridize to the two PLA probes only if they are in close proximity (<40 nm separation), whereas the ligase joins the two hybridized oligonucleotides to form a close circle. Ligation of the oligonucleotides was followed by a rolling‐circle amplification reaction using the ligated circle as a template, resulting in a repeated sequence product. The amplification products were then detected by a fluorescence (Texas Red channel)‐labeled complementary oligonucleotide detection probes. Slides were mounted with Duolink mounting medium containing DAPI nuclear stain. PLA signals (red fluorescent dots) were visualized and imaged using a Leica TCS SP5 confocal microscope.

### Statistical analysis

Collected data were presented as means ± SE. The significance was analyzed using two‐tailed Student's *t* test or two‐way analysis of variance (ANOVA) followed by the Bonferroni post hoc test when more than two treatments were compared. A value of *P *<* *0.05 was considered statistically significant.

## Results

### The role of Orai1 in SOCE and its associated membrane hyperpolarization in VSMCs

**Figure 1:**
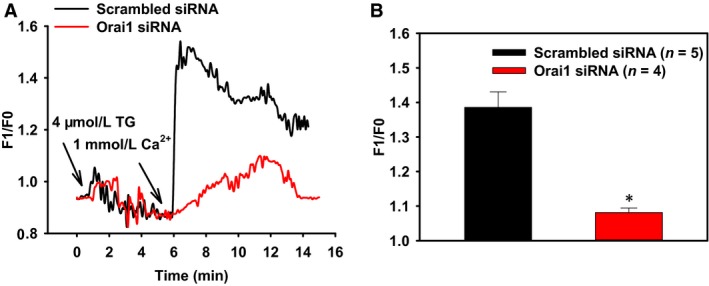
The role of Orai1 in store‐operated Ca^2+^ entry of rat mesenteric artery vascular smooth muscle cells. (A) Representative traces for changes in [Ca^2+^]_i_ in response to thapsigargin (TG) and extracellular Ca^2+^ with the pretreatment of scrambled siRNA or Orai1 siRNA. (B) Summary of data showing changes in [Ca^2+^]_i_ increase in response to extracellular Ca^2+^. Values are means ± SE (*n* = 4–5 samples). *P < 0.05 versus scrambled siRNA.

**Figure 2:**
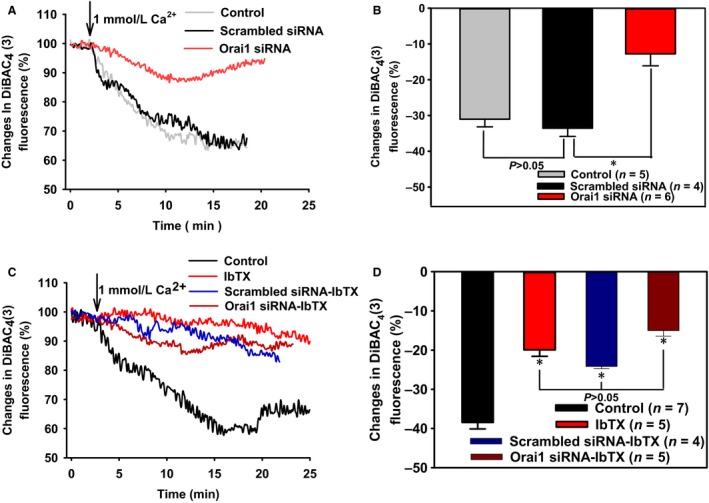
The role of Orai1 and BK_C__a_ in store‐operated Ca^2+^ entry‐induced membrane hyperpolarization of rat mesenteric artery vascular smooth muscle cells (VSMCs). A and C, Representative traces showing after treated with 4 *μ*mol/L thapsigargin for 10 min in 0Ca^2+^‐PSS, membrane hyperpolarization was evoked by 1 mmol/L extracellular Ca^2+^ in VSMCs pretreated with scrambled siRNA or Orai1 siRNA, or without siRNA (control) (A), or with/without 50 nmol/L iberiotoxin (IbTX) (C). B and D, Summary of data showing changes in membrane hyperpolarization in response to extracellular Ca^2+^. Values are means ± SE (*n* = 4–7 samples). *P < 0.05 versus scrambled siRNA or Control.

We first investigated SOCE in the primary cultured VSMCs of rat mesenteric arteries. Preincubation of VSMCs with 4 *μ*mol/L TG for 8–10 min in 0Ca^2+^‐PSS resulted in a rise in [Ca^2+^]_i_, which indicated the Ca^2+^ release from intracellular Ca^2+^ stores. Subsequent addition of extracellular Ca^2+^ (1 mmol/L) initiated SOCE (Figure 1A). The role of SOCE in regulating membrane potential was then examined with a potentiometric fluorescence dye DiBAC_4_(3) (Baczko et al. 2004). Stimulation of SOCE by adding 1 mmol/L extracellular Ca^2+^ resulted in smooth muscle cell membrane hyperpolarization, which was indicated by a significant decrease in DiBAC_4_(3) fluorescence (Figure 2A and 2C). Taken together, our results indicate that SOCE induces membrane hyperpolarization in VSMCs of rat mesenteric arteries.

To explore the functional role of Orai1 in SOCE, we employed RNA interference technique. Orai1 specific siRNA was designed and transfected into primary cultured VSMCs of rat mesenteric arteries. Immunoblotting data confirmed that Orai1 siRNA significantly suppressed Orai1 expression in VSMCs (data not shown). We subsequently used Orai1 siRNA to test the role of Orai1 in [Ca^2+^]_i_ change in VSMCs. Knockdown of Orai1 markedly reduced SOCE compared with the treatment of scrambled siRNA (Figure 1). This result indicates that Orai1 plays an important role in the regulation of SOCE in VSMCs of rat mesenteric arteries.

The effect of Orai1 siRNA on SOCE‐induced membrane hyperpolarization was then examined. Incubation of VSMCs with Orai1 siRNA (1:250) for 24 h significantly inhibited this hyperpolarization compared with the treatment of scrambled siRNA (Figure 2A and 2B), suggesting the pivotal role of Orai1 in modulating SOCE‐associated membrane hyperpolarization. Additionally, scrambled siRNA transfection did not affect SOCE‐induced membrane hyperpolarization compared with control group (Figure 2A and 2B).

### The role of BK_Ca_ in SOCE‐induced membrane hyperpolarization of VSMCs

Presumably, Ca^2+^ influx via Orai1 should depolarize plasma membrane potential instead of hyperpolarization. Therefore, we hypothesized that Ca^2+^ influx via Orai1 may activate BK_Ca_, causing membrane hyperpolarization. A BK_Ca_‐specific blocker IbTX was used to examine this hypothesis. Preincubation of IbTX at 50 nmol/L markedly reduced SOCE‐induced membrane hyperpolarization (Figure 2C and 2D). Note that in the presence of IbTX, Orai1 or scrambled siRNA had no additional effect on SOCE‐induced membrane hyperpolarization, compared with IbTX preincubation alone (Figure 2C and 2D). Taken together, there data strongly suggest that Orai1 is functionally coupled with BK_Ca_ in VSMCs.

### The role of Orai1‐BK_Ca_ coupling in agonist‐induced vasocontraction in rat mesenteric arteries

**Figure 3:**
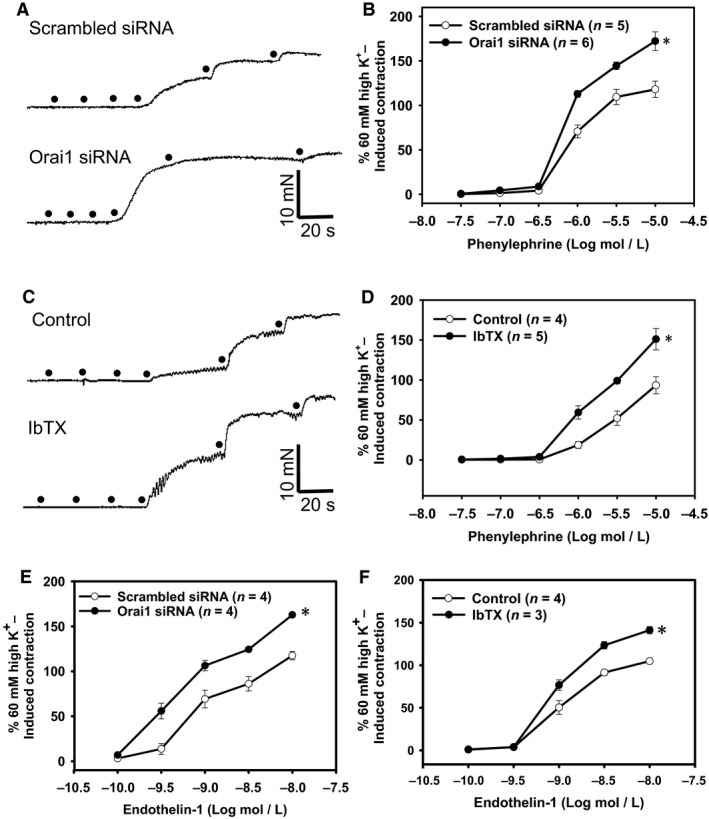
The role of Orai1 and BK_C__a_ in agonist‐induced vasocontraction in rat mesenteric arteries. A and C, Representative traces for phenylephrine (Phe)‐induced concentration‐dependent contraction in rat mesenteric arteries pretreated with scrambled siRNA or Orai1 siRNA (A), or with/without 50 nmol/L iberiotoxin (IbTX) (C). B and D–F, Summarized data showing the effects of Orai1 siRNA (B and E) and IbTX (D and F) on Phe (B and D)‐ or endothelin 1 (E and F)‐induced concentration‐dependent contraction of rat mesenteric arteries. Values are means ± SE(*n* = 3–7 samples). *P < 0.05 versus scrambled siRNA or Control.

We further determined the role of Orai1‐BK_Ca_ coupling in the regulation of agonist‐induced vasocontraction. Our previous study had demonstrated that *α*1‐adrenoceptor agonist Phe and endothelin receptor agonist ET‐1 induced smooth muscle membrane depolarization in isolated rat aortic arteries, accompanied with vasocontraction (Kwan et al. 2009). In this study, our isometric tension results showed that Phe and ET‐1 caused a dose‐dependent vasocontraction in isolated rat mesenteric arteries. Importantly, preincubation of the vessels with Orai1 siRNA (1:250, 24 h) or IbTX (50 nmol/L, 10 min) significantly increased vasocontraction in response to Phe and ET‐1 (Figure 3). These data indicate a role of Orai1‐BK_Ca_ coupling in agonist‐induced vasocontraction in rat mesenteric arteries.

### Orai1 physically associates with BK_Ca_ in smooth muscle of rat mesenteric artery

**Figure 4:**
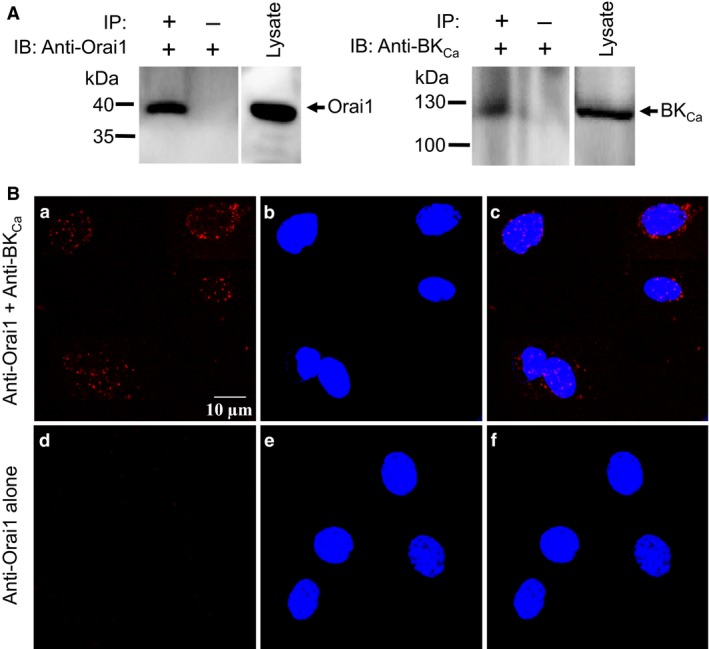
Coimmunoprecipitation and in situ proximity ligation assay of Orai1 and BK_C__a_ in fresh‐isolated rat mesenteric artery smooth muscle cells. A, Representative images showing coimmunoprecipitation followed by immunoblots [left, immunoblot with goat anti‐Orai1 antibody; right, immunoblot with rabbit anti‐BK_C__a_ antibody]. Proteins from smooth muscle layers of rat mesenteric arteries were immunoprecipitated with indicated antibody (+) or preimmune IgG (−). *n* = 3 experiments. B, In situ proximity ligation assay (PLA) analysis to detect the interaction between Orai1 and BK_C__a_. PLA results were displayed in the presence of both goat anti‐Orai1 and rabbit anti‐BK_C__a_ primary antibodies (a–c), or in the presence of goat anti‐Orai1 primary antibody alone (d–f). Duolink secondary antibodies conjugated with oligonucleotides (anti‐goat PLA probe Plus and anti‐rabbit PLA probe Minus) were used to detect primary antibodies. Nuclei (blue) were marked by DAPI staining. Scale bar represents 10 *μ*m.

The above results suggest that Orai1 and BK_Ca_ are functionally coupled. We next tested whether these two proteins are physically associated using a coimmunoprecipitation method. Two antibodies anti‐Orai1 and anti‐BK_Ca_, which are highly specific to their targets respectively, were employed for this experiment. The immunoblot results verified that these two antibodies recognized a single band of Orai1 and BK_Ca_, respectively (Figure 4A). Importantly, coimmunoprecipitation data showed that anti‐BK_Ca_ antibody was able to pull down Orai1 in the protein lysates freshly prepared from smooth muscle layer (Figure 4A, left panel). Moreover, anti‐Orai1 antibody was able to reciprocally pull down BK_Ca_ (Figure 4A, right panel). In control experiments (labeled as IP(‐) in Figure 4A), the pull‐down experiments were conducted using pre‐immune IgG. As expected, no bands were detected (IP(‐) in Figure 4A). Taken together, these results indicate that Orai1 is physically associated with BK_Ca_ to form a signaling complex in smooth muscle of rat mesenteric arteries.

To further confirm that Orai1 and BK_Ca_ indeed have physical interaction, we applied PLA analysis, which detects proteins located within a radius of <40 nm. PLA results in easily detectable fluorescent dots in the presence of both anti‐Orai1 and anti‐BK_Ca_ antibodies in fixed primary cultured VSMCs (Figure 4B: a–c). Moreover, a negative control consisting of incubation with anti‐Orai1 antibody alone displayed a negligible number of fluorescent dots (Figure 4B: d–f). These results suggest that Orai1 indeed physically interacts with BK_Ca_ in rat mesenteric artery VSMCs.

## Discussion

This study demonstrated that in rat mesenteric arteries, (1) SOCE was mediated by Orai1, and Ca^2+^ influx via Orai1 induced membrane hyperpolarization of VSMCs; (2) this Orai1‐mediated membrane hyperpolarization was decreased by BK_Ca_ blocker; (3) inhibition of Orai1 activity by transfection with Orai1‐specific siRNA or preincubation of BK_Ca_ blocker markedly enhanced vasocontraction of rat mesenteric arteries in response to contractile agonists; (4) coimmunoprecipitation data revealed that anti‐Orai1 antibody could pull down BK_Ca_, and anti‐BK_Ca_ antibody could inversely pull down Orai1;(5) PLA analysis showed that Orai1 and BK_Ca_ were physically interacted in VSMCs. Taken together, these results indicate that Orai1 physically interacts with BK_Ca_ to form a signaling complex in rat mesenteric artery VSMCs, and that Ca^2+^ influx via Orai1 activates BK_Ca_, causing membrane hyperpolarization. Furthermore, this hyperpolarizing effect of Orai1‐BK_Ca_ coupling could contribute to suppress contractile agonist‐induced membrane depolarization, preventing excessive contraction of smooth muscle in response to contractile agonists.

Orai1 channel plays an essential role in SOCE. To elucidate the role of Orai1 in smooth muscle of rat mesenteric artery, Orai1 expression was knocked down using specific siRNA. Interestingly, Orai1 siRNA not only effectively suppressed Orai1 protein expression, but also reduced SOCE as well as membrane hyperpolarization. Presumably, suppressing Ca^2+^ influx through Orai1 should result in a decrease in smooth muscle contraction, instead of contraction increase. However, in isometric tension experiments, our data showed that smooth muscle contractility was significantly enhanced after Orai1 proteins were knocked down. Therefore, we hypothesized that Ca^2+^ influx via Orai1 may activate BK_Ca_, leading to a decrease of smooth muscle contraction. BK_Ca_ blocker was used to test this hypothesis. Our data showed that Orai1‐mediated membrane hyperpolarization was decreased by BK_Ca_ blocker, indicating that Orai1‐BK_Ca_ coupling plays an important functional role in regulating SOCE and its associated membrane hyperpolarization in VSMCs of rat mesenteric arteries. Our coimmunoprecipitation and PLA results further demonstrated that Orai1 and BK_Ca_ indeed have physical interaction, which would allow an efficient signal transduction between Orai1 and BK_Ca._

Since both Orai1 and BK_Ca._ are abundantly expressed in many types of smooth muscle cells, we hypothesized that the membrane hyperpolarization initiated by Orai1‐BK_Ca_ coupling may inactivate voltage‐gated Ca^2+^ channels (VGCCs), which dominantly regulate Ca^2+^ influx in VSMCs in response to contractile agonists, thereby contribute to reduce vascular contraction. To test this hypothesis, endogenous contractile agonists Phe and ET‐1 were used, which bind to *α*1‐adrenoceptor and endothelin receptor and induce Ca^2+^ influx via VGCCs, resulting in membrane depolarization and vascular contraction of VSMCs. Additionally, the store Ca^2+^ release was induced by both agonists, which could initiate SOCE. According to the Orai1‐BK_Ca_ coupling model of this study, we suggest that this SOCE may exert its effect to hyperpolarize the plasma membrane and thereby reduce agonist‐mediated membrane depolarization and vasocontraction. Our isometric tension data showed that treatment of the smooth muscle with Orai1 siRNA or BK_Ca_ blocker increased vasocontraction to Phe, indicating that Orai1‐BK_Ca_ coupling is functionally involved in agonist‐induced contraction in VSMCs of rat mesenteric arteries.

Some evidence suggests that K_Ca_ channels could interact with nonvoltage‐gated Ca^2+^ channels to produce a signal transduction between these proteins. TRPC1‐BK_Ca_ coupling contributes to reduce membrane depolarization in response to agonist‐induced vascular contraction, thereby preventing excessive contraction of aortic smooth muscle cells (Kwan et al. 2009). IK_Ca_ physically associates with Orai1 to mediate Ca^2+^ signaling, store refilling and migration in microglia (Ferreira and Schlichter 2013). Our recent study also demonstrated that Orai1 could form a signaling complex with SK3 to control SOCE and its associated membrane hyperpolarization in gallbladder smooth muscle (Song et al. 2015). In this study, we for the first time demonstrated that Orai1 physically interacts with BK_Ca_ in VSMCs of rat mesenteric arteries to regulate muscle contraction. It is possible that live cell may use different signaling complexes such as TRPC1‐BK_Ca_ and Orai1‐BK_Ca_ to response to different agonists. However, even we demonstrated that Orai1 and BK_Ca_ form a signal complex to regulate vascular tone, the role of Orai1‐BK_Ca_ coupling in human disease still remained. The functional change in Orai1‐BK_Ca_ interaction may be linked with some vascular diseases, such as hypertension, diabetes, and atherosclerosis. Therefore, the future study will benefit the understanding of pathological relevance of Orai1‐BK_Ca_ coupling.

In conclusion, we verified that Orai1 physically interacted with BK_Ca_ to form a signaling complex in VSMCs of rat mesenteric arteries. Ca^2+^ influx via Orai1 activates BK_Ca_, causing membrane hyperpolarization. This hyperpolarizing effect of Orai1‐BK_Ca_ could contribute to prevent excessive contraction of smooth muscle in response to contractile agonists.

## Conflict of Interest

None declared.

## References

[phy212682-bib-0001] Baczko, I. , W. R. Giles , and P. E. Light . 2004 Pharmacological activation of plasma‐membrane KATP channels reduces reoxygenation‐induced Ca(2 + ) overload in cardiac myocytes via modulation of the diastolic membrane potential. Br. J. Pharmacol. 141:1059–1067. 14993099 10.1038/sj.bjp.0705702 PMC1574274

[phy212682-bib-0002] Beech, D. J. 2012 Orai1 calcium channels in the vasculature. Pflugers Archiv: European Journal of Physiology 463:635–647. 22402985 10.1007/s00424-012-1090-2 PMC3323825

[phy212682-bib-0003] Berna‐Erro, A. , G. E. Woodard , and J. A. Rosado . 2012 Orais and STIMs: physiological mechanisms and disease. J. Cell Mol. Med. 16:407–424. 21790973 10.1111/j.1582-4934.2011.01395.x PMC3822919

[phy212682-bib-0004] Berridge, M. J. 1998 Neuronal calcium signaling. Neuron 21:13–26. 9697848 10.1016/s0896-6273(00)80510-3

[phy212682-bib-0005] Berridge, M. J. 2013 Dysregulation of neural calcium signaling in Alzheimer disease, bipolar disorder and schizophrenia. Prion 7:2–13. 22895098 10.4161/pri.21767 PMC3609045

[phy212682-bib-0006] Bootman, M. D. , and M. J. Berridge . 1995 The elemental principles of calcium signaling. Cell 83:675–678. 8521483 10.1016/0092-8674(95)90179-5

[phy212682-bib-0007] Chantome, A. , M. Potier‐Cartereau , L. Clarysse , G. Fromont , S. Marionneau‐Lambot , M. Gueguinou , et al. 2013 Pivotal role of the lipid Raft SK3‐Orai1 complex in human cancer cell migration and bone metastases. Cancer Res. 73:4852–4861. 23774210 10.1158/0008-5472.CAN-12-4572

[phy212682-bib-0008] Cheng, K. T. , Y. K. Leung , B. Shen , Y. C. Kwok , C. O. Wong , H. Y. Kwan , et al. 2008 CNGA2 channels mediate adenosine‐induced Ca2 + influx in vascular endothelial cells. Arterioscler. Thromb. Vasc. Biol. 28:913–918. 18292397 10.1161/ATVBAHA.107.148338

[phy212682-bib-0009] Clarysse, L. , M. Gueguinou , M. Potier‐Cartereau , G. Vandecasteele , P. Bougnoux , S. Chevalier , et al. 2014 cAMP‐PKA inhibition of SK3 channel reduced both Ca2 + entry and cancer cell migration by regulation of SK3‐Orai1 complex. Pflugers Archiv: European Journal of Physiology 466:1921–1932. 24458591 10.1007/s00424-013-1435-5

[phy212682-bib-0010] Dominguez‐Rodriguez, A. , I. Diaz , M. Rodriguez‐Moyano , E. Calderon‐Sanchez , J. A. Rosado , A. Ordonez , et al. 2012 Urotensin‐II signaling mechanism in rat coronary artery: role of STIM1 and Orai1‐dependent store operated calcium influx in vasoconstriction. Arterioscler. Thromb. Vasc. Biol. 32:1325–1332. 22223729 10.1161/ATVBAHA.111.243014

[phy212682-bib-0011] Ferreira, R. , and L. C. Schlichter . 2013 Selective activation of KCa3.1 and CRAC channels by P2Y2 receptors promotes Ca(2 + ) signaling, store refilling and migration of rat microglial cells. PLoS ONE 8:e62345. 23620825 10.1371/journal.pone.0062345 PMC3631179

[phy212682-bib-0012] Genazzani, A. A. , and P. Thorn . 2002 Calcium signalling: calcium goes global. Curr. Biol. 12:R432–R433. 12123596 10.1016/s0960-9822(02)00918-1

[phy212682-bib-0013] Gwack, Y. , S. Srikanth , S. Feske , F. Cruz‐Guilloty , M. Oh‐hora , D. S. Neems , et al. 2007 Biochemical and functional characterization of Orai proteins. J. Biol. Chem. 282:16232–16243. 17293345 10.1074/jbc.M609630200

[phy212682-bib-0014] Kwan, H. Y. , Y. Huang , and X. Yao . 2004 Regulation of canonical transient receptor potential isoform 3 (TRPC3) channel by protein kinase G. Proc. Natl Acad. Sci. USA 101:2625–2630. 14983059 10.1073/pnas.0304471101 PMC357000

[phy212682-bib-0015] Kwan, H. Y. , B. Shen , X. Ma , Y. C. Kwok , Y. Huang , Y. B. Man , et al. 2009 TRPC1 associates with BK(Ca) channel to form a signal complex in vascular smooth muscle cells. Circ. Res. 104:670–678. 19168436 10.1161/CIRCRESAHA.108.188748

[phy212682-bib-0016] Li, J. , L. McKeown , O. Ojelabi , M. Stacey , R. Foster , D. O'Regan , et al. 2011 Nanomolar potency and selectivity of a Ca(2)(+) release‐activated Ca(2)(+) channel inhibitor against store‐operated Ca(2)(+) entry and migration of vascular smooth muscle cells. Br. J. Pharmacol. 164:382–393. 21545575 10.1111/j.1476-5381.2011.01368.x PMC3174418

[phy212682-bib-0017] Lin, H. , C. Zheng , J. Li , C. Yang , and L. Hu . 2014 Ca2 + ‐activated K+ channel‐3.1 blocker TRAM‐34 alleviates murine allergic rhinitis. Int. Immunopharmacol. 23:642–648. 25466273 10.1016/j.intimp.2014.10.017

[phy212682-bib-0018] Lipskaia, L. , and A. M. Lompre . 2004 Alteration in temporal kinetics of Ca2 + signaling and control of growth and proliferation. Biol. Cell 96:55–68. 15093128 10.1016/j.biolcel.2003.11.001

[phy212682-bib-0019] Potier, M. , J. C. Gonzalez , R. K. Motiani , I. F. Abdullaev , J. M. Bisaillon , H. A. Singer , et al. 2009 Evidence for STIM1‐ and Orai1‐dependent store‐operated calcium influx through ICRAC in vascular smooth muscle cells: role in proliferation and migration. FASEB J. 23:2425–2437. 19364762 10.1096/fj.09-131128 PMC2717784

[phy212682-bib-0020] Shen, B. , H. Y. Kwan , X. Ma , C. O. Wong , J. Du , Y. Huang , et al. 2011 cAMP activates TRPC6 channels via the phosphatidylinositol 3‐kinase (PI3K)‐protein kinase B (PKB)‐mitogen‐activated protein kinase kinase (MEK)‐ERK1/2 signaling pathway. J. Biol. Chem. 286:19439–19445. 21487005 10.1074/jbc.M110.210294 PMC3103323

[phy212682-bib-0021] Song, K. , X. G., Zhong , X. M. Xia , J. H. Huang , Y. F. Fan , R. X. Yuan , et al. 2015 Orai1 forms a signal complex with SK3 channel in gallbladder smooth muscle. Biochem. Biophys. Res. Commun. 466:456–462. 26367175 10.1016/j.bbrc.2015.09.049

[phy212682-bib-0022] Trebak, M. 2012 STIM/Orai signalling complexes in vascular smooth muscle. J. Physiol. 590:4201–4208. 22641780 10.1113/jphysiol.2012.233353 PMC3473279

[phy212682-bib-0023] Yang, B. , T. Gwozdz , J. Dutko‐Gwozdz , and V. M. Bolotina . 2012 Orai1 and Ca2 + ‐independent phospholipase A2 are required for store‐operated Icat‐SOC current, Ca2 + entry, and proliferation of primary vascular smooth muscle cells. Am. J. Physiol. Cell Physiol. 302:C748–C756. 22094335 10.1152/ajpcell.00312.2011 PMC3774541

[phy212682-bib-0024] Zhang, W. , K. E. Halligan , X. Zhang , J. M. Bisaillon , J. C. Gonzalez‐Cobos , R. K. Motiani , et al. 2011 Orai1‐mediated I (CRAC) is essential for neointima formation after vascular injury. Circ. Res. 109:534–542. 21737791 10.1161/CIRCRESAHA.111.246777 PMC3164514

